# Association of D-dimer and Fibrinogen With Hypercoagulability in COVID-19 Requiring Extracorporeal Membrane Oxygenation

**DOI:** 10.1177/0885066621997039

**Published:** 2021-03-01

**Authors:** Abhimanyu Chandel, Saloni Patolia, Mary Looby, Najeebah Bade, Vikramjit Khangoora, Christopher S. King

**Affiliations:** 1 Department of Pulmonology and Critical Care, 8395 Walter Reed National Military Medical Center, Bethesda, MD, USA; 2 6886 Virginia Commonwealth University School of Medicine, Richmond, VA, USA; 3 Department of Pharmacy, 23146 Inova Fairfax Hospital, Falls Church, VA, USA; 4 Department of Hematology, 23146 Inova Fairfax Hospital, Falls Church, VA, USA; 5 Department of Advanced Lung Disease and Transplant, 23146 Inova Fairfax Hospital, Falls Church, VA, USA

**Keywords:** COVID-19, venous thromboembolism, extracorporeal membrane oxygenation, thromboelastography

## Abstract

**Background:**

D-dimer concentration has been used by institutions to identify candidates for intensified anticoagulant treatment for venous thromboembolism prevention and for the mitigation of the microthrombotic complications associated with COVID-19. Thromboelastography (TEG) maximum amplitude (MA) has been validated as a marker of hypercoagulability and MA ≥68 mm has been utilized as a marker of hypercoagulability in other conditions.

**Methods:**

The goal of this study was to evaluate the relationship between coagulation, inflammatory, and TEG parameters in patients with COVID-19 on extracorporeal membrane oxygenation (ECMO). We performed a single-center retrospective analysis of consecutive patients that received ECMO for the treatment of COVID-19. TEG, inflammatory, and coagulation markers were compared in patients with and without a thrombotic complication. Correlation tests were performed to identify the coagulation and inflammatory markers that best predict hypercoagulability as defined by an elevated TEG MA.

**Results:**

A total of 168 TEGs were available in 24 patients. C-reactive protein and fibrinogen were significantly higher in patients that developed a thrombotic event versus those that did not (*P* = 0.04 and *P* = 0.04 respectively). D-dimer was negatively correlated with TEG MA (*P* < 0.01), while fibrinogen was positively correlated (*P* < 0.01). A fibrinogen >441 mg/dL was found to have a sensitivity of 91.2% and specificity of 85.7% for the detection of MA ≥68 mm.

**Conclusions:**

In critically ill patients with COVID-19 treated with ECMO, D-dimer concentration had an inverse relationship with degree of hypercoagulability as measured by TEG MA. D-dimer elevation may potentially reflect hemostatic perturbation in patients on ECMO or the severity of COVID-19 related sepsis rather than designate patients likely to benefit from anticoagulation. Fibrinogen concentration may represent a more useful marker of hypercoagulability in this population.

## Introduction

Critically ill coronavirus disease 2019 (COVID-19) patients have been noted to display features of hypercoagulopathy involving both microthrombosis and macrothrombotic events. These patients have frequently been observed to have significant laboratory abnormalities that are compatible with a hypercoagulable state including severe derangements of D-dimer, fibrinogen, and thromboelastography (TEG) parameters.^1,2^

Thrombosis, including venous thromboembolism (VTE), arterial thrombosis, and microthrombosis, is thought to be a sequelae of the pathogenesis of severe COVID-19 infection. Autopsies of COVID-19 infected patients have demonstrated the presence of pulmonary microthrombi and it has been postulated that microthrombus formation may contribute to end-organ dysfunction and failure.^3–5^

One approach to the management of critically ill patients with COVID-19 has been the use of therapeutic anticoagulation to mitigate this hypercoagulability. Multiple local and regional organizations have advocated for the use of D-dimer testing and threshold values as a means to guide the intensity of anticoagulant therapy.^6,7^ This practice is based in part on observational evidence suggesting that treatment of patients with COVID-19 with anticoagulation is associated with improved in-hospital survival.^8^ Additionally, D-dimer elevation independently correlates to risk of VTE in general medical patients, and furthermore, correlates to mortality in COVID-19.^9,10^

However, D-dimer is a non-specific marker that is produced as the result of fibrin formation and degradation as a downstream product of thrombin cleavage of fibrinogen.^11^ Although elevated D-dimer levels are observed with the presence of VTE, D-dimer is non-specific and often markedly elevated in other conditions including sepsis in the absence of clinically evident VTE.^12^ It is not clear that the magnitude of D-dimer elevation in COVID-19 correlates with hypercoagulability or clinical conditions that are likely to benefit from anticoagulation.^2^ It is also not clear that D-dimer represents a superior marker for hypercoagulability over other available tests of the coagulation system or inflammatory markers including fibrinogen, ferritin, and C-reactive protein (CRP). TEG maximum amplitude (MA), a measure of clot strength accounting for platelet and fibrin contributions, has been validated as a marker of hypercoagulability in a wide range of conditions and has been found to correlate with macrothrombotic events.^13^ Previous studies have defined hypercoagulability by a TEG MA ≥68 mm.^14^ In this study we evaluated the relationship between commonly utilized laboratory parameters and hypercoagulability as represented by TEG MA in patients on extracorporeal membrane oxygenation as part of the management of COVID-19.

## Materials and Methods

We performed a retrospective medical record review of consecutive hospitalized patients admitted to Inova Fairfax Hospital, a tertiary care referral center in Falls Church, Virginia, USA, between March 5, 2020, and May 29, 2020, with a diagnosis of COVID-19 confirmed by positive PCR testing and undergoing extracorporeal membrane oxygenation (ECMO). The study was approved by the Institutional Review Board (IRB# 18-3317) at Inova Fairfax Hospital. Informed consent was not required by the Institutional Review Board given the research involved no more than minimal risk to the study subjects.

Only patients on ECMO were evaluated as it is standard practice at our institution to obtain daily TEGs as part of the routine care of these patients. TEG was performed using the TEG 5000 platform (Haemonetics Corporation, Braintree, MA). TEGs were conducted using citrated whole blood, kaolin activator, and heparinase for patients on heparin. D-dimer values are reported by our laboratory in ug/mL of fibrinogen equivalent units (FEU).

Data were abstracted in a structured format by one of the authors (SP) and entered into a customized data form. Data was collected when available for standard TEG parameters (R-time and MA), D-dimer, fibrinogen, CRP, and ferritin. D-dimer values >20 ug/mL FEU were recorded as 20 ug/mL FEU and R-time reported as >60 min was denoted as 60 min. TEG and inflammatory markers or coagulation studies were compared if drawn within 24 hours. For patients on systemic heparin, TEG parameters with the use of heparin neutralization were collected. Demographic data, anticoagulant usage, and clinical outcomes including bleeding, macrothrombotic complications after the initiation of ECMO, and time on ECMO were recorded. Diagnosis of a macrothrombotic event (deep vein thrombosis, pulmonary embolism, or other large vessel venous or arterial blood clot) was recorded based on radiographic report finalized during hospitalization or point-of-care ultrasound findings consistent with VTE in patients with acute decompensation and suspected pulmonary embolism. Bleeding was defined as a clinically observed event, as reported by the treating clinician, that necessitated transfusion of red blood cells.

### Statistical Analysis

Distribution of all continuous data was examined for normality using visual inspection and the Wilk Shapiro test. Laboratory characteristics of the groups are presented as the median and interquartile range and compared using the Wilcoxon rank sum test. Categorical data are presented as counts with proportions and compared using Fisher’s exact test (2-tailed). A *P* value <0.05 was considered statistically significant.

MA, fibrinogen, and CRP had skewed distributions. To reduce the impact of outliers and to satisfy the principle assumptions of parametric modeling, the natural log of these variables is used in graphical presentations and analyses. Associations of continuous data are presented graphically using linear regression models and plotted using natural log (ln) scales. MA values were categorized into values ≥68 mm and <68 mm and the diagnostic accuracy of fibrinogen and CRP in predicting MA results is presented using a receiver operator characteristic curve, summarized using the area under the curve (AUC) together with 95% CIs, and compared utilizing the empirical method. Univariate and multivariate logistic regression analyses of factors possibly associated with macrothrombosis were performed. Clinical and laboratory parameters found to be correlated to macrothrombosis in univariate analysis were entered into the model (*P* < 0.1). All statistical analyses were performed using STATA version 14 (StataCorp LP; College Station, TX, USA).

## Results

Twenty-four consecutive patients with COVID-19 requiring venovenous ECMO support were included in the final cohort. Patients were grouped by the presence or absence of diagnosed macrothrombosis after ECMO initiation. Macrothrombosis was identified in 12 patients while receiving ECMO or after this therapy was discontinued (9 deep vein thromboses, 2 pulmonary emboli, and 1 episode of embolic mesenteric ischemia). Two patients developed bleeding complications (hemoptysis and gastrointestinal hemorrhage). Demographic data, median laboratory values, and clinical outcomes between groups are displayed in Table 1. Patients with imaging confirmed macrothrombosis were significantly more likely to have higher median CRP and fibrinogen values.

**Table 1.** Characteristics of Patients Categorized by Diagnosed Macrothrombosis.^a^

	All subjects	Macrothrombosis	No thrombosis	*P* value
	N = 24	N = 12	N = 12	
Age (years)	46 (37, 53)	50 (41, 52)	45 (26, 54)	0.37
Gender, women	4 (16.7)	0 (0)	4 (33.0)	0.09
Race, non-white	20 (83.3)	11 (91.7)	9 (75.0)	0.59
Time prior to ECMO initiation (days)	5 (2, 7)	6 (2, 9)	5 (2, 6)	0.40
MA (mm)	72.8 (71.2, 78.5)	74.9 (72.2, 80.2)	72.1 (70.4, 78.1)	0.23
R time (min)	10.4 (8.3, 12.8)	11.6 (8.4, 12.6)	10.3 (8.1, 13.4)	0.86
D-dimer (ug/mL)	3.5 (2.3, 8.0)	3.1 (2.3, 5.5)	3.9 (2.4, 9.5)	0.31
Fibrinogen (mg/dL)	543.5 (474.0, 690.3)	582.5 (537.8, 768.8)	501.5 (443.0, 578.3)	0.04
CRP (mg/dL)	11.1 (4.8, 17.8)	15.3 (8.1, 27.0)	7.6 (2.8, 12.8)	0.04
Ferritin (ng/mL)	985.2 (623.0, 2070.5)	1004.6 (532.5, 2536.5)	965.8 (654.9, 2070.5)	0.92
Bleeding complication	2 (8.3)	2 (16.7)	0 (0)	0.48
Time on ECMO (days)	13 (9, 21)	16 (11, 23)	10 (7, 19)	0.11
Death during hospitalization	5 (20.8)	4 (33.3)	1 (8.3)	0.32

Abbreviations: MA, maximum amplitude; CRP, C-reactive protein.

^a^ Data are presented as median (25th percentile, 75th percentile) or n (%) unless otherwise indicated.

All patients studied received therapeutic anticoagulation per institutional ECMO protocol with occasional cross-over between specific anticoagulant. Forty-six TEGs were obtained on heparin, while 122 TEGs were obtained on bivalirudin. Matching D-dimer values were available for all TEGs and there were 7 instances where matching fibrinogen values were not obtained.

**Figure 1.**
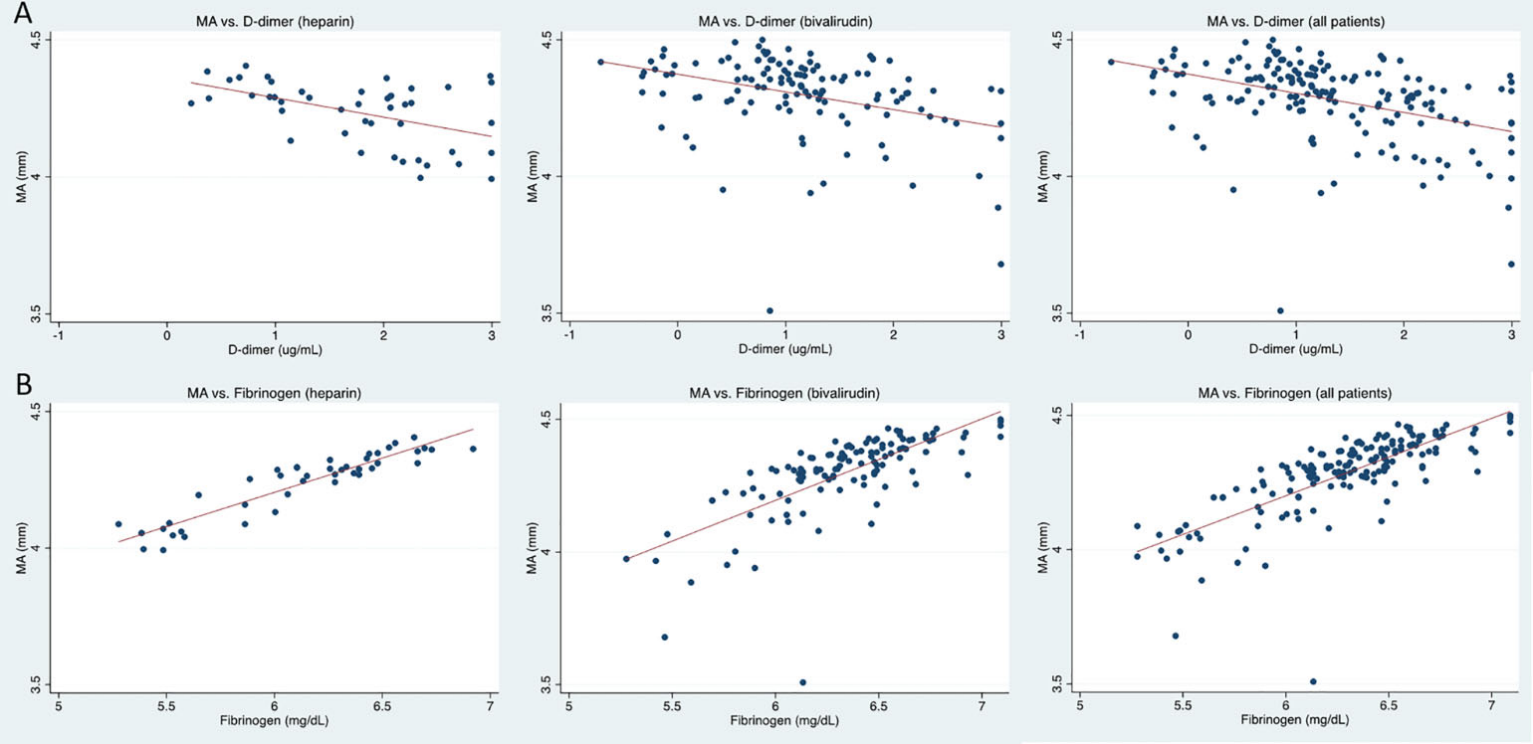
(A) Ln (MA) vs. ln(D-dimer) with linear regression for patients on heparin, bivalirudin, and aggregated patients. (B) Ln (MA) vs. ln(fibrinogen) with linear regression for patients on heparin, bivalirudin, and aggregated patients.

D-dimer was examined as a possible predictor of MA in patients on heparin, on bivalirudin, and in aggregate. D-dimer was found to be significantly negatively correlated with MA in all 3 analyses (*P* < 0.01) (Figure 1A). D-dimer explained little of the variance in MA values (adjusted R^2^ = 0.22, 0.11, and 0.16 respectively). Fibrinogen was found to be positively correlated with MA. Ln(fibrinogen) explained over 50% of the variance in ln(MA) (adjusted R^2^ = 0.57) and the association was significant for patients on heparin, bivalirudin, and in aggregate (*P* < 0.01) (Figure 1B). With ln(fibrinogen) in the model, CRP and ferritin were examined as additional predictors and were not significant.

**Figure 2.**
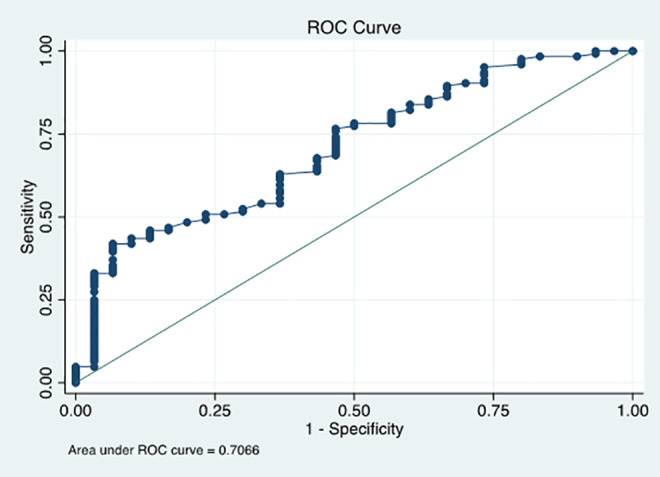
ROC curve for C-reactive protein as a predictor of maximum amplitude ≥68 mm 11x8mm (1200 x 1200 DPI).

**Figure 3.**
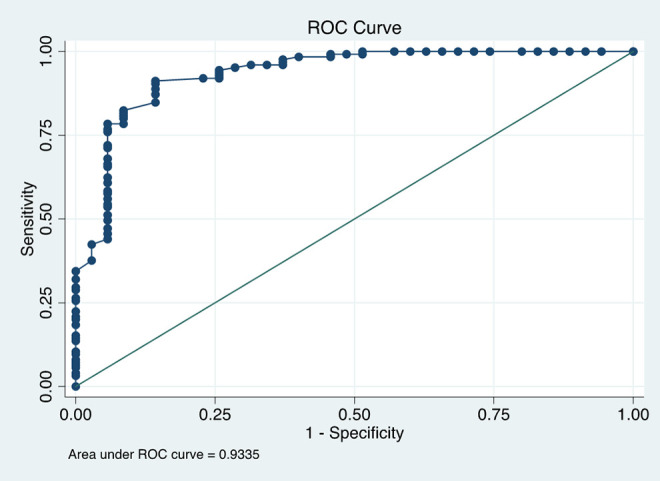
ROC curve for fibrinogen as a predictor of maximum amplitude ≥68 mm 22x16mm (1200 x 1200 DPI).

All TEG MA values were then categorized as ≥68 mm and <68 mm. MA values ≥68 mm were associated with lower absolute D-dimer values and higher absolute fibrinogen values (*P* < 0.01). Receiver operator curves (ROC) based on CRP (Figure 2) and fibrinogen levels (Figure 3) were estimated. CRP accuracy in classifying MA ≥68 mm was poor (AUC 0.71 CI: 0.61-0.81), while classification accuracy of fibrinogen for a MA ≥68 mm was excellent (AUC 0.93 CI: 0.88-0.98). AUC was greater for the ROC curve utilizing fibrinogen as a predictor (*P* < 0.01). A fibrinogen cutoff of 441 mg/dL to predict MA ≥68 mm (sensitivity of 91.2%, specificity 85.7%) satisfies the closest-to-(0, 1) criterion for threshold selection.

Demographic and laboratory parameters were examined with logistic regression to identify factors predictive of macrothrombosis. CRP and fibrinogen were predictive of thrombosis in univariate analysis. In multivariate analysis, only median CRP was found to be an independent predictor of the development of macrothrombosis (*P* = 0.04; OR 3.23, 95% CI 1.03-10.12).

## Discussion

The emergence of COVID-19 as a global pandemic has necessitated the implementation of treatment protocols based on expert opinion given limited available clinical data. D-dimer has been cited as correlating with worse clinical outcomes in COVID-19 and coagulopathy has been noted to be a common feature of severe infection.^3–5,10^ However, absolute magnitude of D-dimer has not been convincingly demonstrated to be linked to an increased risk of small or large vessel thrombosis in this disease.^2^

In the present study, we aimed to evaluate the correlation of commonly available measures of coagulopathy (D-dimer and fibrinogen) with a validated reference standard (TEG). TEG MA values have previously been demonstrated to correlate with degree of hypercoagulability in a wide number of clinical conditions.^14^

Our results demonstrate that in this population of critically ill COVID-19 patients receiving ECMO therapy, D-dimer did not correlate positively with degree of hypercoagulability as measured by TEG MA. On the contrary; D-dimer concentration was actually inversely related to MA in our study population. This finding was unexpected and the physiological basis is uncertain. A possible explanation for this phenomenon may be that given D-dimer is a byproduct of fibrinolysis and MA in part measures the strength of a fully formed clot; it would stand to reason that higher levels of fibrinolysis may reflect weaker clot strength and, thus, create the inverse relationship noted in this study.

A number of national guidelines have been published regarding the management of COVID-19 coagulopathy. These guidelines, in our view, are appropriately conservative with respect to recommendations related to the use of therapeutic dosing of anticoagulants for the management of critically ill patients with COVID-19.^2,3,15^ Despite these guidelines, local and regional hospital practices vary widely, with some recommending D-dimer thresholds to aid the decision regarding initiation and intensity of anticoagulation. Based on our data, we speculate that commonly noted severe D-dimer aberrations may potentially reflect COVID-19 related sepsis and the associated imbalance between intravascular and extravascular fibrin formation in patients at risk of delayed clearance of D-dimer given systemic illness rather than solely represent the degree of risk of macrothrombosis. Given that hemostatic perturbations are common in ECMO, further prospective study is indicated to investigate the relationship between D-dimer, TEG parameters, and hypercoagulability in patients with COVID-19 who are not being managed with this therapy.

Studies have noted a clear association between fibrinogen and MA values in other clinical conditions.^14^ Marked derangements in fibrinogen has been noted to be common in COVID-19 and an analysis of patients with COVID-19 not receiving ECMO and studied at entry into the ICU also demonstrated an association between MA and fibrinogen.^10,16^ In this study, fibrinogen levels were independent predictors of clot strength among patient with other signs of hypercoagulable status.^16^ We found fibrinogen to be a much better predictor of MA than D-dimer. In our population a fibrinogen of greater than 441 mg/dL had sensitivity of 91.2% and specificity 85.7% for detection of MA ≥68 mm. This finding is of interest given that TEG is not routinely available at many centers and takes specialized training to perform to achieve accurate results. Fibrinogen is a commonly available laboratory tests in most hospital environments and results may be easier to obtain. Thus, it may represent a more practical method for the identification of patients at risk for COVID-19 related hypercoagulability. A new viscoelastic device, the VCM (not yet available in the US but certified in Europe), may offer point-of-care bedside thromboelastograph results which may be useful, especially in austere environments, where fibrinogen testing is not easily available.^17^

Other investigators have compared hemostatic changes in COVID-19 to those noted in disseminated intravascular coagulation (DIC) related to sepsis. Unlike DIC, fibrinogen clotting activity in COVID-19 is frequently elevated. Further, coagulation factors such as prothrombin time and activated partial thromboplastin time do not follow the typical pattern usually seen with DIC related consumptive coagulopathy.^18^ The physiological basic of the hypercoagulable state noted in patients with COVID 19 is not well proven. We postulate that an imbalance between procoagulant factors and anticoagulant factors is logical. How elements such as factor VIII, antithrombin, von Willebrand factor, and others explain the observed hypercoagulability has yet to be fully elucidated.

Our findings should be interpreted in the context of the study limitations. First, our data are retrospectively derived from a single tertiary care center. The study was limited to severe COVID-19 infection requiring ECMO support due to the institutional use of TEG as part of the standard care of these patients. Notably, patients were not universally screened for VTE prior to the initiation of ECMO. Of the patients included in this study, none underwent cross-sectional imaging prior to ECMO initiation and only 4 patients underwent formal doppler ultrasonography to exclude the presence of deep vein thrombosis (median 3.5 days prior to ECMO initiation). Therefore, it cannot be stated with certainty that VTE identified in patients on ECMO did not develop earlier in the hospital course. Further, alterations in platelet function is a known phenomenon in patients on ECMO and the implications of this alteration may affect TEG, inflammatory, and coagulation parameters.^19^ Microvesicles are particles derived from platelets or monocytes and carry procoagulant activity. Microvesicles have been associated with VTE and have also been noted in patients on ECMO.^20^ The interplay between COVID-19 disease and the effects of ECMO may also increase the risk of thromboembolism in these patients. Further, clinical thrombosis is also influenced by the degree of fibrinolysis and clot breakdown in addition to coagulation and platelet contributions. Degree of fibrinolysis may be best represented by the TEG parameter LY30 (which reflects clot lysis at 30 minutes following MA).^21^ However, this parameter was not available for the TEG samples analyzed in this study as the standard protocol for TEG performance at our institution during the study period was to stop testing prior to the 30 minutes required to report this value. Given these considerations, our selected population may limit generalizability and our findings should be validated in a non-ECMO cohort. Second, all patients in our study were on therapeutic anticoagulation with either heparin or bivalirudin per standard institutional ECMO protocol. Deviation from protocolized dosing of therapeutic anticoagulation was occasionally noted. Several of the patients in this report experienced clotting at cannulation sites despite boluses of heparin and one patient received tissue plasminogen activator in addition to anticoagulation for repetitive pulmonary emboli. There is limited data regarding the use of bivalirudin in patients with COVID-19 on ECMO and further investigation of direct thrombin inhibitors versus traditional heparin anticoagulation is needed. The role of antiplatelet agents should also be explored further in this population. As a result, the effects of anticoagulation and dosing adjustments of these medications on the study outcomes are unknown and may introduce a source of bias. Of note, all findings were consistent when examined for patients on heparin compared to patients on bivalirudin. Finally, although TEG has been validated as a useful marker of hypercoagulability in other conditions; MA has not been sufficiently evaluated in COVID-19 and is a surrogate marker for more important clinical outcomes of hypercoagulability such as macro and microthrombosis. Our study lacks suitable sample size to detect the ability of MA, CRP, D-dimer, or fibrinogen to discriminate these clinical outcomes related to hypercoagulability in COVID-19. Likewise, a recent study where computed tomography was performed within 4 days of ECMO decannulation estimated the incidence of VTE to be greater than 70%. A significant proportion of these clots occurred within the inferior vena cava, findings which may be missed by lower extremity doppler ultrasonography.^22^ The incidence of VTE in our cohort (50%) was less than this prior literature, which could reflect underdiagnosis of VTE in our population. Thus, while fibrinogen and CRP demonstrated a significant increase in patients with macrothrombosis in our cohort, further prospective study is necessary to better define this relationship.

In critically ill patients with COVID-19 treated with ECMO, D-dimer appears to have an inverse relationship with degree of hypercoagulability as measured by TEG MA. D-dimer elevation in this setting may potentially reflect the severity of COVID-19 related sepsis or hemostatic perturbation in patients on ECMO rather than designate patients at risk for macrothrombotic complications. Fibrinogen concentration may represent a more useful marker of hypercoagulability in this population and further study is warranted.
